# Reshifting Na^+^ from Shoots into Long Roots Is Associated with Salt Tolerance in Two Contrasting Inbred Maize (*Zea mays* L.) Lines

**DOI:** 10.3390/plants12101952

**Published:** 2023-05-11

**Authors:** Zhenyang Zhao, Hongxia Zheng, Minghao Wang, Yaning Guo, Yingfei Wang, Chaoli Zheng, Ye Tao, Xiaofeng Sun, Dandan Qian, Guanglong Cao, Mengqian Zhu, Mengting Liang, Mei Wang, Yan Gong, Bingxiao Li, Jinye Wang, Yanling Sun

**Affiliations:** 1 School of Marine Science and Engineering, Qingdao Agricultural University, Qingdao 266237, China; zzy16678184420@163.com (Z.Z.); emiya1518@163.com (M.W.); gyn1506718741@163.com (Y.G.); 18404214325@163.com (Y.W.); zhengchaoli99@163.com (C.Z.); yet2625468938@163.com (Y.T.); sunxiaofeng1028@163.com (X.S.); d2556726502@163.com (D.Q.); cao20001023@163.com (G.C.); openblood@163.com (M.Z.); 18706557502@163.com (M.L.); 17860839878@163.com (M.W.); 17703698334@163.com (Y.G.); lbx3081984076@163.com (B.L.); wangjy519@vip.163.com (J.W.); 2 Key Laboratory of Saline-Alkali Vegetation Ecology Restoration, Ministry of Education, College of Life Sciences, Northeast Forestry University, Harbin 150040, China; zhenghongxia2020@foxmail.com

**Keywords:** maize seedlings, long root, salt tolerance, Na^+^ redistribution, *ZmHAK1*, *ZmNHX1*

## Abstract

Maize, as a glycophyte, is hypersensitive to salinity, but the salt response mechanism of maize remains unclear. In this study, the physiological, biochemical, and molecular responses of two contrasting inbred lines, the salt-tolerant QXH0121 and salt-sensitive QXN233 lines, were investigated in response to salt stress. Under salt stress, the tolerant QXH0121 line exhibited good performance, while in the sensitive QXN233 line, there were negative effects on the growth of the leaves and roots. The most important finding was that QXH0121 could reshift Na^+^ from shoots into long roots, migrate excess Na^+^ in shoots to alleviate salt damage to shoots, and also improve K^+^ retention in shoots, which were closely associated with the enhanced expression levels of *ZmHAK1* and *ZmNHX1* in QXH0121 compared to those in QXN233 under salt stress. Additionally, QXH0121 leaves accumulated more proline, soluble protein, and sugar contents and had higher SOD activity levels than those observed in QXN233, which correlated with the upregulation of *ZmP5CR*, *ZmBADH*, *ZmTPS1*, and *ZmSOD4* in QXH0121 leaves. These were the main causes of the higher salt tolerance of QXH0121 in contrast to QXN233. These results broaden our knowledge about the underlying mechanism of salt tolerance in different maize varieties, providing novel insights into breeding maize with a high level of salt resistance.

## 1. Introduction

High salinity levels represent one of the major environmental threats to crop yields in agriculture. It has been estimated that up to 20% of cultivated land and 50% of irrigated land are affected by salt stress worldwide [1]. High salinity levels could disturb the tissue water status and ion balance in plants; induce cell membrane structure and lipid changes; affect the activity of major cytosolic enzymes, photosynthesis and utilization of mineral nutrition [2,3,4,5]; inhibit plant germination; and trigger tissue burning, leaf abscission, and root cell death, which cause serious production losses [6]. Thus, we must clarify the underlying mechanism of salt tolerance in plants and improve crop production.

Plant resistance to high levels of salinity is complex and comprises a multitude of processes, including diverse gene, hormonal, and stomatal regulation, ion transport and osmotic adjustment; the production of compatible solutes; and the activation of the antioxidant defense system [7,8]. In resistance to high Na^+^ concentrations, plants could activate the exclusion of Na^+^ and/or its sequestration into the intracellular vacuole [6,7]. It has previously been reported that plants’ responses to salt stress have two phases. These are osmotic stress in the early phase, when several physiological and biochemical indexes of shoots and roots are initially affected and plant growth inhibition and tissue damage occur. Ionic stress occurs in the later phase, in which Na^+^ toxicity accumulates in tissues and disrupts K^+^ homeostasis, affecting important cellular processes [4]. A relatively low Na^+^ concentration and a concurrent high K^+^ concentration in the cytosol are important to maintaining the activities of the enzymes involved in metabolism [9,10]. Many studies in a model plant *Arabidopsis thaliana* and its mutants have uncovered the regulated mechanisms of salt tolerance, including the salinity overly sensitive (SOS) signaling pathway, the calcium signaling pathway, mitogen-activated protein kinase cascades, and reactive oxygen species (ROS) homeostasis [11,12]. In crop plants, many salt-responsive proteins have been reported, such as vacuolar Na^+^/H^+^ antiporters (NHXs), high-affinity K^+^ transporters (HKTs), and SOSs, which have important roles in the regulation of Na^+^ and K^+^ ion balance [13,14,15].

Na^+^ and K^+^ homeostasis is crucial for the salt tolerance of plants. A previous study on many *Arabidopsis* ecotypes demonstrated that a strong capacity for K^+^ retention depended on the plants’ high salinity tolerance [6]. In maize, salt tolerance in two contrasting inbred lines was dependent on the ability to remove Na^+^ and retain K^+^ [10]. Acclimation could improve the salt stress tolerance of maize due to a high level of K^+^ retention in plants [16]. In plants, many K^+^ transporter families have been reported, and KT (K^+^ transporter)/HAK (high-affinity K^+^)/KUP (K^+^ uptake) was the largest family among them [17]. In maize, ZmHAK1, which belongs to this family, has an important role in K^+^ distribution in shoots [18]. Additionally, ZmHAK4 was shown to promote Na^+^ exclusion in shoots, and ZmHAK5 partakes in K^+^ uptake in roots [18,19]. In addition, the HKT family is critical in Na^+^ and K^+^ homeostasis in plants [20,21], and sequence variations of HKT transporters have been shown to be associated with salt tolerance [15,19,22,23]. In maize, ZmHKT1 promotes leaf Na^+^ exclusion from the xylem sap and maintains a low ROS level to enhance salt tolerance in maize [24,25]. Multiple NHXs have been identified in crop plants, which function in the accumulation of Na^+^ in vacuoles, K^+^ homeostasis, pH regulation, and cell expansion under high levels of salinity [14,26].

Plants can accumulate many compatible solutes to alleviate salt damage, including proline, glycine betaine (GB), and sugars [8]. Proline is accumulated to mediate osmotic adjustment and protect the enzyme activities in plant tissues [27,28,29], and pyrroline-5-carboxylate reductase (P5CR) and pyrroline-5-carboxylate synthase (P5CS) are key enzymes, which encode enzymes involved in the proline biosynthesis pathway [6,30]. GB is accumulated to enhance ion balance and protect the subcellular structures under salt stresses, and betaine aldehyde dehydrogenases (BADH) are responsible for its biosynthesis [31,32]. Trehalose is a nonreducing diglucoside elevated in plants under salt stress, probably protecting protein and membrane structures and regulating the antioxidant systems to ameliorate ROS scavenging, and trehalose-6-phosphate synthase (TPS) is one of the key enzymes in terms of trehalose biosynthesis [33]. Moreover, in maize, glycine betaine [34,35], trehalose [36], ascorbate–proline–glutathione [37], and phosphate [28], are applied to counter salt stress, which is favorable for the maintenance of Na^+^/K^+^ balance and improvements in antioxidant defense. Recently, single or combined analyses of genomes [38], transcriptomes [34,39], proteomes [40,41,42,43], and metabolomes [44,45] and a genome-wide association study (GWAS) revealed differential expression genes, small RNAs, and regulatory networks in plants under salt stress [46,47,48], which provided valuable information to understand the mechanism of salt response.

In the present study, two contrasting inbred maize lines, named QXN233 and QXH0121, were investigated in terms of Na^+^ and K^+^ distribution, changes in compatible solutes, and antioxidant enzyme activity as well as the expression of relevant genes between them to uncover their physiological and molecular differences in response to salt stress.

## 2. Results

### 2.1. Salt Tolerance Differences between QXN233 and QXH0121 Inbred Lines

**Figure 1:**
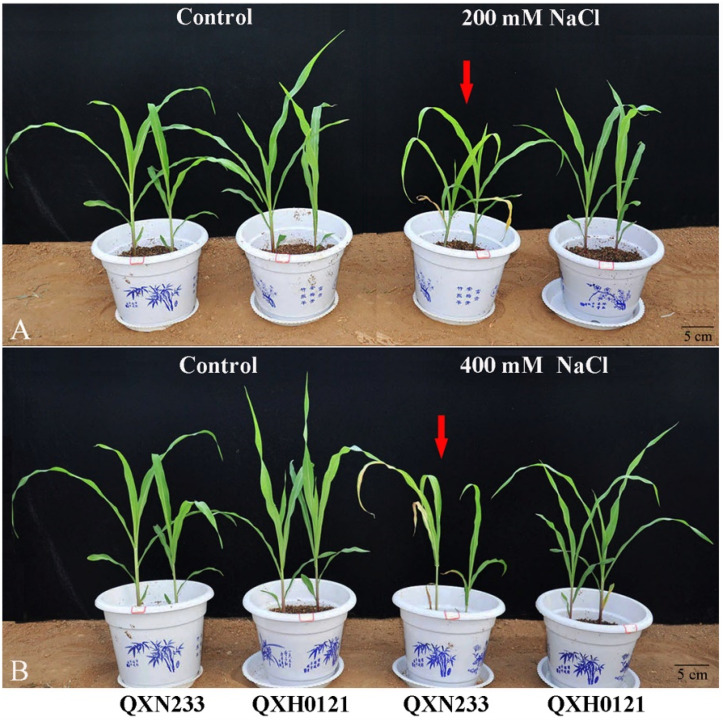
Phenotypic responses of QXN233 and QXH0121 to salt stress. The plant growth of QXN233 and QXH0121 when exposed to 200 mM NaCl (**A**) and 400 mM NaCl (**B**) stress for 10 d using a pot culture assay. The red arrow indicates that the leaves of QXN233 were withered. Bar = 5 cm.

Obviously, in this study, QXN233 was salt-sensitive, and QXH0121 was salt-tolerant. As shown in Figure 1, QXH0121 seedlings remained green and normal, while QXN233 emerged with severe leaf-wilting and stunted growth (Figure 1, Table 1). In QXN233, old leaves mostly became injured and new leaves were also wilted (Figure 1, the red arrow). Increasing chlorosis emerged with long-term salinity, and old leaves were more rapidly damaged when exposed to higher salinity levels (Figure 1B and Figure S1). Significantly, QXH0121 exhibited stronger root growth than QXN233 (Figure S2; Table 1). Similar phenotypes were also observed in QXN233 and QXH0121 using a hydroponics assay (Figure S3).

**Figure 2:**
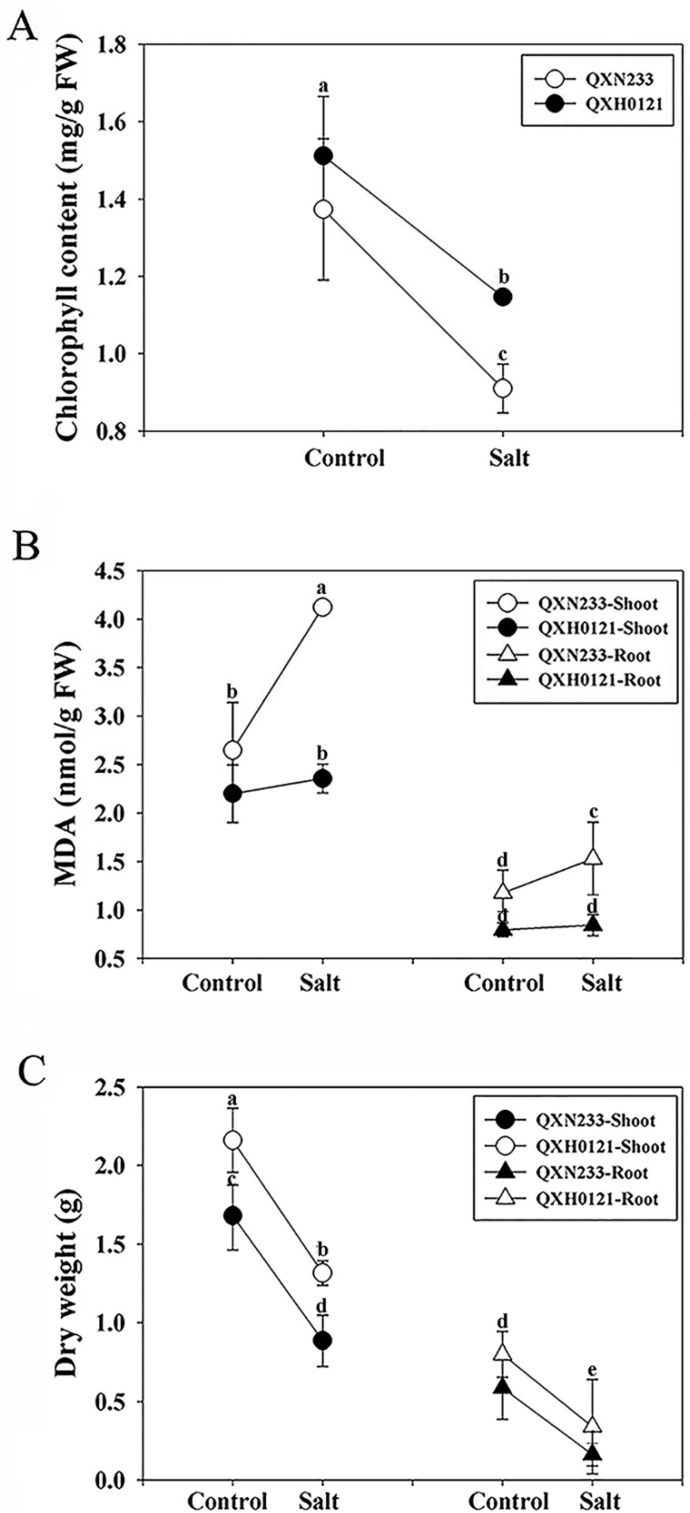
Changes in physiological indexes in QXN233 and QXH0121 under salt stress. QXN233 and QXH0121 seedlings were exposed to 200 mM NaCl stress for 30 d. Chlorophyll content (**A**), MDA content (**B**), and dry weight (**C**) were measured in QXN233 and QXH0121. Values represent means ± SE, and different letters indicate significant differences in QXH0121 vs. QXN233 (LSD test, *p* < 0.05).

Several important physiological indexes were investigated in QXN233 and QXH0121 responding to salinity. Under salt stress, the chlorophyll contents both decreased, with lower content in QXN233 than in QXH0121 (Figure 2A). The shoots and roots of QXN233 accumulated higher MDA contents than those of QXH0121 (Figure 2B). In addition, the dry weight of QXN233 seedlings was reduced severely in comparison to QXH0121 under salt stress (Figure 2C).

### 2.2. Na^+^ and K^+^ Changes in QXN233 and QXH0121

**Figure 3:**
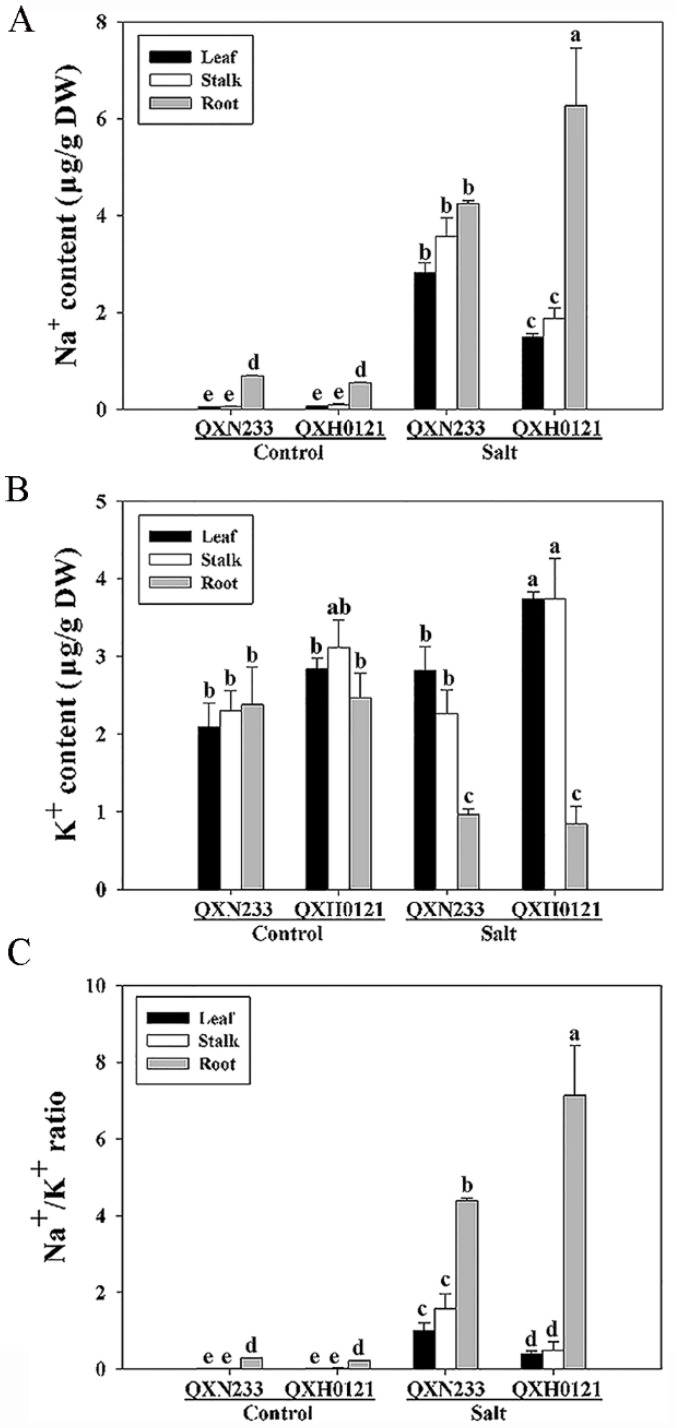
Changes in Na^+^ and K^+^ ion contents in QXN233 and QXH0121. QXN233 and QXH0121 seedlings were exposed to 200 mM NaCl stress for 30 d. Na^+^ contents (**A**) and K^+^ contents (**B**) of QXN233 and QXH0121 were measured in leaves, stalks, and roots under salt stress, and Na^+^/K^+^ ratios (**C**) of them were analyzed. Values represent means ± SE, and different letters indicate significant differences in QXH0121 vs. QXN233 (LSD test, *p* < 0.05).

Na^+^ and K^+^ contents were measured in the leaves, stalks, and roots of QXN233 and QXH0121. The results showed that the Na^+^ contents were increased in both QXN233 and QXH0121 seedlings under salt stress (Figure 3A). Significantly, high Na^+^ contents were detected in QXH0121 roots, and less Na^+^ accumulated in leaves and stalks under salt stress, suggesting that QXH0121 could transfer more Na^+^ to its long roots. In contrast, there were no differences in the roots of QXN233 in comparison to the aboveground parts (Figure 3A, right). Under salt stress, QXH0121 had more K^+^ redistribution in its leaves and stalks than that of QXN233 (Figure 3B, right). Correspondingly, QXH0121 had a low Na^+^/K^+^ ratio in its leaves and stalks in comparison to QXN233, supporting its high tolerance (Figure 3C). Therefore, it was concluded that in QXH0121, there was a strong Na^+^ shift from shoots into roots to induce salt tolerance.

### 2.3. Alterations in Compatible Solutes in QXN233 and QXH0121

**Figure 4:**
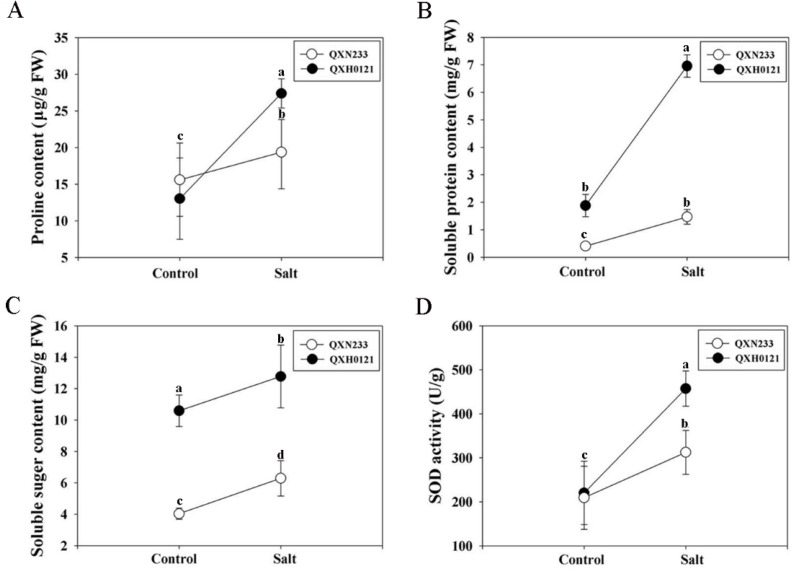
Quantitative analyses of proline content (**A**), soluble protein content (**B**), soluble sugar content (**C**), and superoxide dismutase (SOD) activity (**D**) of QXN233 and QXH0121 leaves under salt stress. QXN233 and QXH0121 seedlings were exposed to 200 mM NaCl stress for 30 d. Values represent means ± SE, and different letters indicate significant differences in QXH0121 vs. QXN233 (LSD test, *p* < 0.05).

The rapid accumulation of three compatible solutes (proline, soluble protein, and soluble sugar) was examined in QXN233 and QXH0121 after a long-term salt treatment. Significantly, the proline content in QXH0121 increased by two-fold compared to that of QXN233 under salt stress (Figure 4A). Additionally, under salt stress, the accumulations of soluble protein and sugar were both notably higher in QXH0121 than in QXN233, although high background levels existed in QXH0121 (Figure 4B,C). In particular, the soluble protein contents of QXH0121 increased by more than seven-fold compared to QXN233 (Figure 4B). In addition, SOD activity was more enhanced in QXH0121 than in QXN233 under salt stress (Figure 4D).

### 2.4. Expression Patterns of Na^+^ and K^+^ Ion Transporter Genes in QXN233 and QXH0121

**Figure 5:**
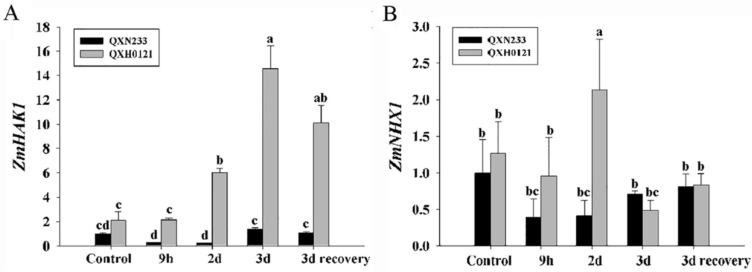
Expression analysis of *ZmHAK1* and *ZmNHX1* genes in QXN233 and QXH0121 leaves under salt stress. The QXN233 and QXH0121 seedlings were exposed to 50 mM NaCl stress at 0 h, 9 h, 2 d, and 3 d and recovered to normal conditions for 3 d using a hydroponics assay, and the expression of two genes, *ZmHAK1* (**A**) and *ZmNHX1* (**B**), in leaves of them, were examined via quantitative real-time PCR. Values represent means ± SE, and different letters indicate significant differences in QXH0121 vs. QXN233 during the time of the experiment (LSD test, *p* < 0.05).

*ZmHAK1* was increasingly upregulated in QXH0121 leaves from 9 h to 3 d under salinity, and -it slightly decreased in the 3 days of normal recovery after the salt treatment (3 d recovery). A similar expression pattern emerged in QXN233 roots, except a decrease was seen on day 3 under salinity (Figure S4A). The expression level of *ZmHAK1* in QXH0121 leaves and roots was remarkably higher than in QXN233 at all salt treatment times (Figure 5A and Figure S4A). Under salt stress, *ZmNHX1* in leaves was more upregulated in QXH0121 than in QXN233 on day 2 under salinity, despite the fact that no significant change emerged at other time points (Figure 5B). The expression level of *ZmNHX1* in the roots of QXH0121 showed no difference from that in QXN233 9 h and 2 d under salinity, but a higher expression level of *ZmNHX1* was shown in QXH0121 compared to QXN233 on day 3 and in the 3 d recovery period (Figure S4B).

### 2.5. Expression Patterns of Some Salt-Responsive Genes in QXN233 and QXH0121

**Figure 6:**
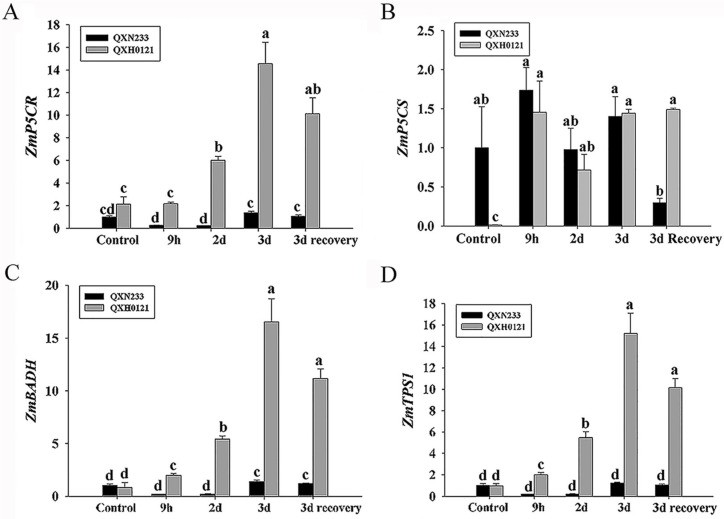

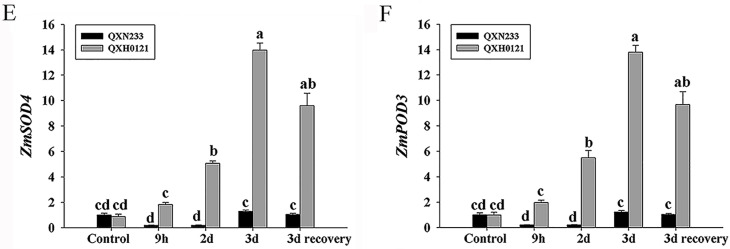
Expression analysis of *ZmP5CR, ZmP5CS, ZmBADH*, *ZmTPS1*, *ZmSOD4*, and *ZmPOD3* genes in QXN233 and QXH0121 leaves under salt stress. The QXN233 and QXH0121 seedlings were exposed to 50 mM NaCl stress at 0 h, 9 h, 2 d, and 3 d recovered to normal conditions for 3 d using a hydroponics assay. In leaves, the *ZmP5CR* (**A**), *ZmP5CS* (**B**), *ZmBADH* (**C**), *ZmTPS1* (**D**), *ZmSOD4* (**E**), and *ZmPOD3* (**F**) genes were examined via quantitative real-time PCR. Values represent means ± SE of three replicates, and different letters indicate significant differences in QXH0121 vs. QXN233 during the time of the experiment (LSD test, *p* < 0.05).

In leaves, *ZmP5CR* was significantly increased in QXH0121 compared to QXN233 under the salinity and 3 d recovery conditions. *ZmP5CS* was upregulated in QXH0121, but no different from that of QXN233 under salinity, despite a high native level in QXH0121 relative to QXN233 (Figure 6A,B). Similarly, *ZmBADH*, *ZmTPS1*, *ZmSOD4*, and *ZmPOD3* were all obviously upregulated in QXH0121 leaves compared to that of QXN233 leaves, especially at the peak of 3 d under salinity (Figure 6C–F). In roots, these four genes, except for *ZmP5CR* and *ZmP5CS*, showed higher expression levels in QXH0121 than in QXN233 on day 2 under salt stress, and the levels almost recovered to normal levels after 3 d of recovery (Figure S6).

## 3. Discussion

Soil salinization, as one of the major limiting factors in agriculture, has great effects on crop growth and production [1,6,10]. Previously, many reports focused on plant adaptation to salt stress [2,4,6,23]. However, the mechanism of salt tolerance remains unclear due to its complexity and the multiple factors involved [10,49]. In the present study, the salt-tolerant QXH0121 leaves remained green with high chlorophyll contents, while QXN233 leaves became withered, especially old leaves, probably due to excessive Na^+^ retention, as has previously been reported [9]. Remarkably, QXH0121 had longer root lengths than QXN233 under the salinity condition. It has been previously reported that roots act as the main sites for plants sensing salt stress and determine the whole plant’s production capacity [50]. Thus, this largely led to a high biomass in QXH0121 compared to that in QXN233. In addition, MDA content is a product of lipid peroxidation and is regarded as a marker of membrane damage under various types of stress [12]. QXH0121 had lower MDA content than QXN233, implying reduced membrane damage in QXH0121 [7,27,51].

Na^+^ and K^+^ ion homeostasis is a key factor for salt tolerance in plants, as has been previously reported [3,6,9,10,18,21,24,25]. Various strategies are adopted in plants to relieve Na^+^ toxins, such as reducing Na^+^ uptake, sequestering Na^+^ into vacuoles, recycling Na^+^ from the xylem stream to the roots and expelling Na^+^ from roots [2,6,9,12,52]. In different plants, the salt tolerance of barley varieties was attributed to lower Na^+^ retention in their shoots than that of the salt-sensitive rice varieties, which was consistent with their high expression levels of the *SOS*, *HKT*, and *NHX* genes under salt stress [52]. In two maize genotypes with arbuscular mycorrhizal (AM) fungi inoculation, the salt tolerance was improved by accelerating the Na^+^ shoot-to-root translocation rate and mediating Na^+^/K^+^ distribution between shoots and roots, associated with the expression of the ion transporter genes *ZmSOS1*, *ZmHKT1* and *ZmNHX* [53]. Supporting previous reports, in this study, the salt-tolerant QXH0121 line accumulated less Na^+^ content in shoots and translocated more Na^+^ into its long roots, whereas in the salt-sensitive QXN233 line, no distinct Na^+^ redistribution was seen in the whole plant. As has been reported, Na^+^ and K^+^ have similar physicochemical properties and a competitive binding site between them [9,54]. Correspondingly, QXH0121 possessed more K^+^ in leaves and stalks and a lower Na^+^/K^+^ ratio than that of QXN233, probably due to more Na^+^ being recycled to its long roots. It has been previously reported that ZmHAK1 took part in K^+^ distribution in the shoots of maize [18]. Consistent with the previous report that *ZmHAK1* was responsible for K^+^ distribution in shoots, it was always significantly increased during the salt treatment and peaked at 3 d under salt stress and was still high during 3 d recovery period in QXH0121 compared with QXN233. These results suggest that QXH0121 had a better ability to transfer K^+^ into the aboveground parts than QXN233. Additionally, *ZmNHX1* was significantly upregulated to some degree in QXH0121 vs. QXN233 under salt stress, mediating its Na^+^ regulation and ion balance in plant cells [13,55]. In the future, many other salt-sensitive and -tolerant varieties should be investigated in terms of their Na^+^ and K^+^ distribution and the expression patterns of ion transporters. Additionally, many other salt-responsive genes, such as *SOS1*, *HKT1*/*2*, *HAK4*/*5*, *MYB*, and *CIPK*, need to be investigated between QXH0121 and QXN233 [15,17,18,19,22,23,25].

Compatible solutes, including amino acids, sugars, glycine betaine, and other low molecular-weight metabolites, were increased in plants to re-establish osmotic homeostasis under detrimental environmental factors [2,7,8,9,27,32,41]. In this study, three compatible solutes (proline, soluble protein and soluble sugar) were all increased in QXN0121 and QXN233 under salt stress, in agreement with previous reports [31,32,33]. Proline is a common osmoregulatory compound that protects enzymes and reduces ROS damage [8,56]. *ZmP5CR* and *ZmP5CS* were shown to be two key enzyme genes in proline synthesis [57]. Compared to QXN233, QXH0121 accumulated high proline contents and had an increased expression level of *ZmP5CR* rather than *ZmP5CS*, suggesting that *ZmP5CR* might be a key cause of the different proline contents between them. A previous study on metabolic contributions to salt stress in two maize hybrids showed that an accumulation of sugars (glucose, fructose, and sucrose) in leaves was identified as a salt-resistance adaptation [44]. Glycine betaine and trehalose could be helpful for maintaining ion balance and protecting membrane integrity and antioxidant enzyme activities [34,35,36]. *ZmBADH* and *ZmTPS1*, which encode the key enzymes for glycine betaine and trehalose biosynthesis, were both upregulated in QXH0121, consistent with its higher soluble sugar contents than QXN233. In addition, QXH0121 had higher soluble protein contents relative to QXN233 under salt stress. QXH0121 had abundant soluble protein and sugar contents compared to QXN233 under control conditions, which was related to their different genetic backgrounds. Moreover, QXH0121 exhibited higher SOD activity levels than QXN233 due to a high expression level of *ZmSOD4* in QXH0121. Additionally, *ZmPOD3* was obviously upregulated in QXH0121 compared to QXN233, implying high levels of POD activity in QXH0121, which needs to be confirmed in the future. In all, QXH0121 had high compatible solutes contents and SOD activities, which enabled it to scavenge more ROS and reduce MDA and are associated with the salt tolerance of QXH0121. Recently, comparative transcriptomic and proteomic analysis of two maize inbred lines showed that the salt-tolerant line could maintain the osmotic regulation ability, the synergistic effects of antioxidant enzymes, energy supply capacity, signal transduction, ammonia detoxification ability, lipid metabolism, and nucleic acid synthesis [41,58]. Significantly, in the future, transcriptomic and proteomic metabolomic analyses between the salt-tolerant QXH0121 and the salt-sensitive QXN233 could be performed to reveal more key genes or metabolites related to their salt tolerance.

## 4. Materials and Methods

### 4.1. Plant Growth and NaCl Treatments

QXN233 and QXH0121, two maize genotypes, have previously been reported [59]. The seeds from them were surface-sterilized with a 10% sodium hypochlorite (NaClO) solution for 10 min, rinsed three times with sterilized water, and germinated at 28 °C in the dark between two layers of wet filter paper in sterile Petri dishes in an artificial culture chamber. After germination, the normal seedlings were transferred to foam boards with 5 L of modified normal Hoagland’s nutrient solution, and its composition was as follows: 25 mΜ H_3_BO_4_, 2 mM Ca(NO_3_)_2_, 0.65 mM MgSO_4_, 0.5 mM KH_2_PO_4_, 50 μM KCl, 25 μM Fe-EDTA, 5 μM MnSO_4_, 2 μM ZnSO_4_, 0.5 μM CuSO_4_, and 0.005 μM (NH_4_)_6_Mo_7_O_24_. The fresh nutrient solution was replaced every day. In the greenhouse, the light/dark cycle was 14/10 h, the temperature was at 26 ± 4 °C, and the relative humidity was controlled at 60–65%.

Using a pot culture assay, seedlings at the three-leaf stage were transferred to pots filled with vermiculite and then covered with nutrient solution containing 0, 200, and 400 mM NaCl for 10 and 30 d. Then, 0.5 L of the same fresh solution was applied every two days. Then, the samples were harvested and photographed.

Using a hydroponic assay, seedlings at the three-leaf stage were transferred to containers with 0.3 L of nutrient solution containing 50 mM NaCl for 0 h, 9 h, 2 d, and 3 d. After NaCl treatment, the seedlings were immediately transferred to the normal nutrient solution for 3 d of recovery. Then, the samples were harvested and photographed.

### 4.2. Growth Parameters, Chlorophyll, and Malondialdehyde (MDA) Content

At the three-leaf stage, the seedlings were treated with 200 mM NaCl for 30 d using a pot culture assay. The plant height, leaf width, and the length of the longest leaf and root length were measured using a dividing rule. Then, the fresh leaves and roots were fixed at 105 °C for 30 min and dried at 85 °C for 3 d. Finally, the dry weights (DWs) of the leaves and roots were weighed.

Meanwhile, the same parts of leaves in QXN233 and QXN0121 were collected, and their chlorophyll contents were measured using the ethanol extraction method [7], and the MDA contents of the leaves and roots in them were measured using the thiobarbituric acid assay method [60].

### 4.3. Na^+^ and K^+^ Contents

At the three-leaf stage, the seedlings were treated with 200 mM NaCl for 30 d using a pot culture assay. The leaves, stalks, and roots were collected and weighed, and then, the Na^+^ and K^+^ contents were analyzed via atomic absorption spectrometry (PE-2100; Perkin Elmer Corporation, Wellesley, MA, USA) and an inductively coupled plasma spectrometer (Optima 7300 DV; Perkin Elmer Corporation, Wellesley, MA, USA), respectively. The presented results are the average and standard error of standard error (SE) for three biological replicates. At least six plants were included in each of the three replicate measurements.

### 4.4. Proline, Soluble Sugar, Soluble Protein Content and Superoxide Dismutase (SOD) Activity

At the three-leaf stage, the seedlings were treated with a 200 mM NaCl solution for 30 d using a pot culture assay. The leaf and root tissues were collected and weighed. Then, the proline contents of them were measured via a ninhydrin-based colorimetric assay [7]. The soluble sugar contents were measured using the anthrone–sulfuric acid method [28], the soluble protein contents were determined using the dying method with Coomassie brilliant blue [28], and SOD activities were measured via nitroblue tetrazolium photoreduction [60]. The presented results are the average and standard error of SE for three biological replicates. At least six plants were included in each of the three replicate measurements.

### 4.5. Quantitative Real-Time PCR (qRT-PCR) Analysis

Total RNA samples were extracted from the leaves and roots of NaCl-treated maize seedlings with the E.Z.N.A Plant RNA Kit (Omega Bio-tek, Norcross, GA, USA), using a hydroponic assay as described in Section 2.1. cDNA was synthesized with 5× All-in-One RT MasterMix (AccuRT Genomic DNA Removal Kit included) (ABM, Vancouver, BC, Canada) and amplified with the Ultra SYBR Mixture kit (DBI Bioscience, Germany) on an ABI 7500 Real-time PCR system (ABI, USA). The *18S RNA* gene was used as an internal control, and *ZmHAK1* (GRMZM2G093826), *ZmNHX1* (AY270036.1) *ZmP5CR* (DQ026301), *ZmP5CS* (DQ864376), *ZmBADH* (EU019896), *ZmTPS1* (AF529266), *ZmSOD4* (XM_008650839), and *ZmPOD3* (GRMZM2G427815) were examined. The data were analyzed using the 2^−∆∆Ct^ method. The genes and the primers used in this study are listed in Table S1. Each qRT-PCR was based on three biological replicates and three technical replicates.

### 4.6. Statistical Analysis

Three biological replicates were applied for all of the above experiments, and the data were reported as mean values ± SE. All of the data obtained were subjected to an analysis of variance (ANOVA), and the significant differences between genotypes and treatments were compared via the least-significant difference (LSD) test at the *p*-value (*p* < 0.05) indicated by different lowercase letters.

## 5. Conclusions

In this study, the salt sensitivity and salt tolerance of two maize inbred lines, QXN233 and QXH0121, were investigated, respectively. Under salt stress, QXH0121 had higher chlorophyll content and dry weight and lower MDA content than QXN233. Importantly, QXH0121 possessed lower Na^+^ content in its leaves and stalks and higher Na^+^ content in its long roots, contributing to its salt tolerance. However, QXN233 did not display such distinct Na^+^ redistribution. Correspondingly, QXH0121 showed an ability to acquire more K^+^ in shoots under salt stress and exhibited a lower Na^+^/K^+^ ratio than QXN233, which was associated with its high transcript levels of *ZmHAK1* and *ZmNHX1*. In addition, QXH0121 accumulated high proline, soluble protein, and sugar levels, as well as increased SOD activity levels compared to QXN233, which correlated with its high transcript levels of *ZmP5CR*, *ZmBADH*, *ZmTPS1*, and *ZmSOD4*. These results support that the QXH0121 line is more salt tolerant than the QXN233 line, which is associated with its efficient Na^+^ ion redistribution and high levels of compatible solutes, as well as its improved ability to scavenge antioxidants.

## Figures and Tables

**Table 1:** Quantitative analyses of growth indexes in QXN233 and QXH0121 under salt stress. QXN233 and QXH0121 were treated with 200 mM NaCl and 400 mM NaCl for 30 d, respectively. Plant height, leaf width and leaf length of the longest leaf, and root length of QXN233 and QXH0121 were measured. Values represent means ± SE of three replicates. Different letters indicate a significant difference between the two genotypes (LSD test, *p* < 0.05).

Plants	Treatments	Plant Height (cm)	Leaf Width (cm)	Leaf Length (cm)	Root Length (cm)
QXN233	Control	59.75 ± 0.30 ^a^	2.65 ± 0.30 ^a^	50.75 ± 0.30 ^a^	41.00 ± 0.90 ^b^
	200 mM NaCl	37.25 ± 5.50 ^c^	1.85 ± 0.10 ^b^	43.00 ± 5.00 ^b^	18.25± 1.50 ^d^
QXH0121	Control	46.00 ± 4.20 ^b^	1.95 ± 0.07 ^ab^	56.00 ± 1.40 ^a^	60.00± 4.50 ^a^
	200 mM NaCl	34.25 ± 0.30 ^c^	1.80 ± 0.04 ^b^	43.00 ± 1.70 ^b^	40.00 ± 2.40 ^c^
QXN233	Control	58.95 ± 0.34 ^a^	2.75 ± 0.36 ^a^	51.75 ± 0.26 ^a^	40.00 ± 0.67 ^b^
	400 mM NaCl	35.15 ± 3.40 ^c^	1.60 ± 0.14 ^b^	40.12 ± 4.90 ^b^	17.85 ± 1.34 ^d^
QXH0121	Control	45.69 ± 3.68 ^b^	1.97 ± 0.11 ^ab^	55.79 ± 1.10 ^a^	59.46± 4.34 ^a^
	400 mM NaCl	34.75 ± 0.29 ^c^	1.67 ± 0.13 ^b^	42.19 ± 1.15 ^b^	39.14 ± 3.16 ^c^

## Data Availability

Data are contained within the article or Supplementary Materials.

## Supplementary Materials

The following supporting information can be downloaded at: https://www.mdpi.com/article/10.3390/plants12101952/s1, Figure S1: Phenotypic responses of QXN233 and QXH0121 to salt stress; Figure S2: Root responses of QXN233 and QXH0121 to salt stress; Figure S3: Phenotypic responses of QXN233 and QXH0121 to salt stress; Figure S4: Expression analysis of ZmHAK1 and ZmNHX1 genes in QXN233 and QXH0121 roots under salt stress; Figure S5: Expression analysis of ZmP5CR, ZmP5CS, ZmBADH, ZmTPS1, ZmSOD4 and ZmPOD3 genes in QXN233 and QXH0121 roots under salt stress. Table S1: Primers used in quantitative real-time PCR.
