# Supplementary material for: Reshifting Na+ from Shoots into Long Roots Is Associated with Salt Tolerance in Two Contrasting Inbred Maize (Zea mays L.) Lines
Source: Plants (Basel). 2023 May 11;12(10):1952. doi: 10.3390/plants12101952 (PMC10220590; doi:10.3390/plants12101952)
Supplement: Supplementary file 1 [file plants-12-01952-s001.zip › plants-2227556-supplementary.pdf]

Supplementary Information (SI)
Original Research Article:

Reshifting Na^+ from Shoots into Long Roots Is Associated with Salt Tolerance in Two Contrasting Inbred Maize (*Zea mays* L.) Lines

Zhenyang Zhao [1,†], Hongxia Zheng [2,†], Minghao Wang [1,†], Yaning Guo [1,†], Yingfei Wang [1], Chaoli Zheng [1], Ye Tao [1], Xiaofeng Sun [1], Dandan Qian [1], Guanglong Cao [1], Mengqian Zhu [1], Mengting Liang [1], Mei Wang [1], Yan Gong [1], Bingxiao Li, Jinye Wang [1] and Yanling Sun [1,*]

[1] School of Marine Science and Engineering, Qingdao Agricultural University, Qingdao 266237, China; zzy16678184420@163.com (Z.Z.); emiya1518@163.com (M.W.); gyn1506718741@163.com (Y.G.); 18404214325@163.com (Y.W.); zhengchaoli99@163.com (C.Z.); yet2625468938@163.com (Y.T.); sunxiaofeng1028@163.com (X.S.); d2556726502@163.com (D.Q.); cao20001023@163.com (G.C.); openblood@163.com (M.Z.); 18706557502@163.com (M.L.); 17860839878@163.com (M.W.); 17703698334@163.com (Y.G.); lbx3081984076@163.com (B.L.); wangjy519@vip.163.com (J.W.)

[2] Key Laboratory of Saline-Alkali Vegetation Ecology Restoration, Ministry of Education, College of Life Sciences, Northeast Forestry University, Harbin 150040, China; zhenghongxia2020@foxmail.com

* Correspondence: 201901116@qau.edu.cn; Tel.: +86-0532-8655-0511

† These authors contributed equally to this work.

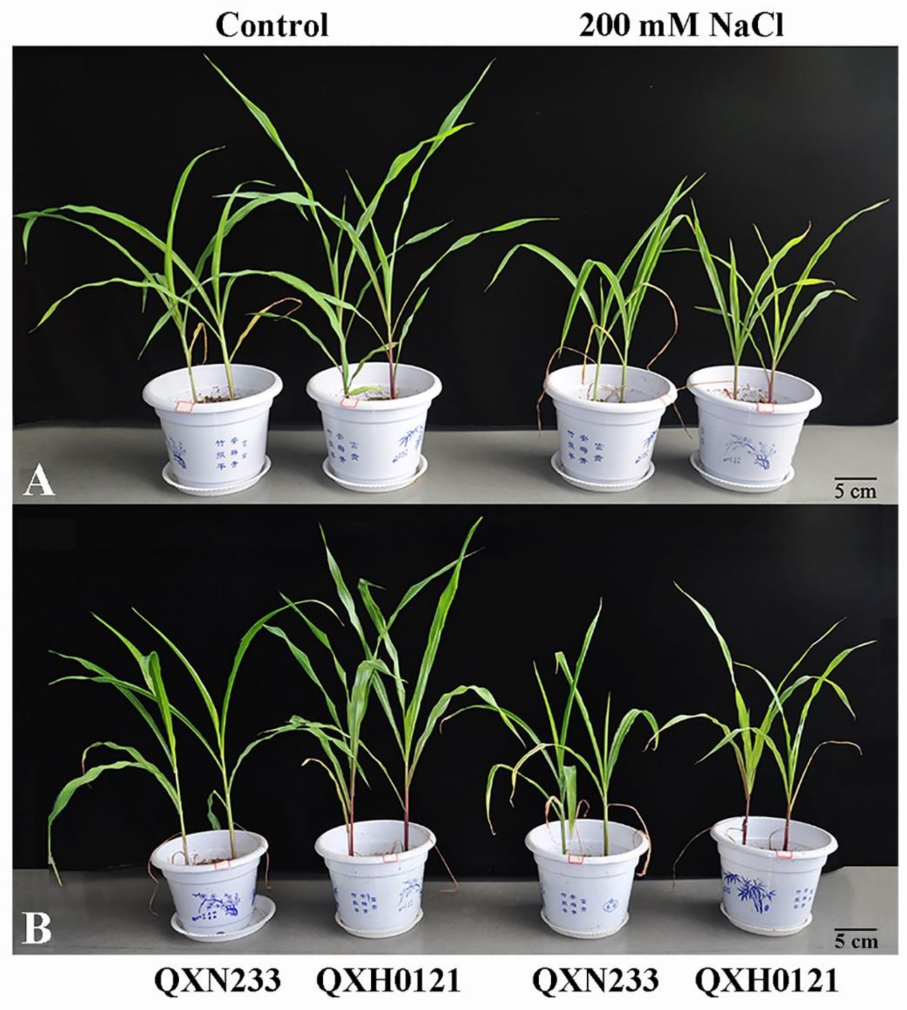

Figure S1. Phenotypic responses of QXN233 and QXH0121 to salt stress. QXN233 and QXH0121 seedlings were exposed to 200 mM NaCl stress for 20 d (A) or 30 d (B) using a pot culture assay. Bar = 5 cm.

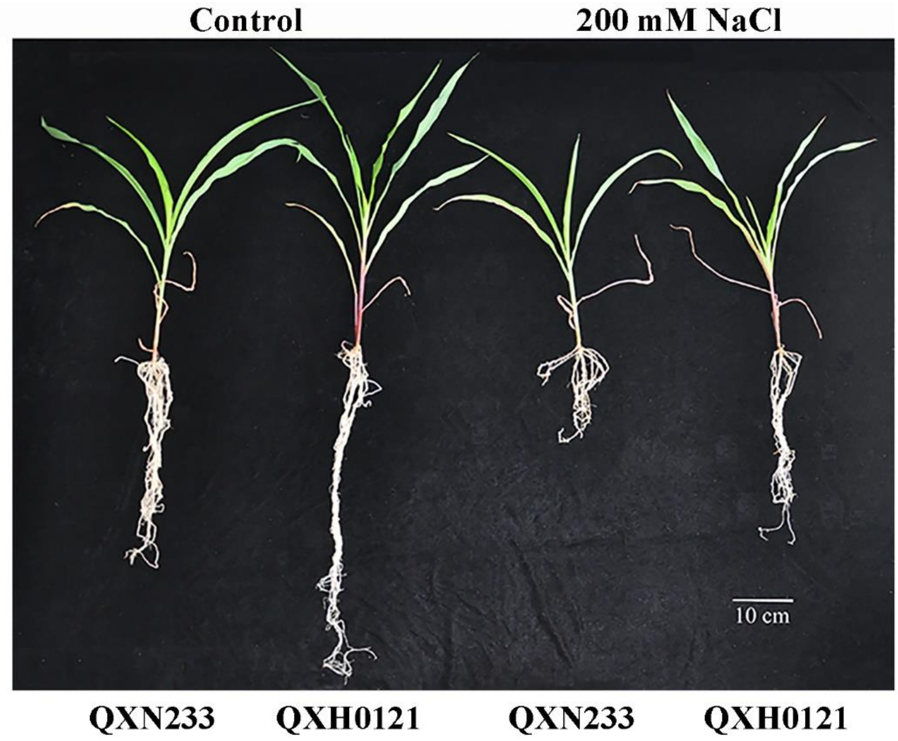

Figure S2. Root responses of QXN233 and QXH0121 to salt stress. QXN233 and QXH0121 seedlings were exposed to 200 mM NaCl stress for 20 d. Bar = 10 cm.

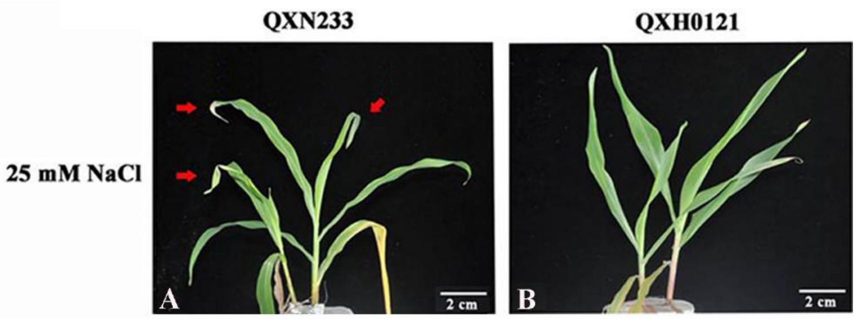

Figure S3. Phenotypic responses of QXN233 and QXH0121. QXN233 and QXH0121 seedlings were exposed to 25 mM NaCl stress for 5 d using a hydroponics assay. Phenotypes ofQXN233 (A) and QXH0121 (B) were recorded and took photos. The red arrows indicated the withered leaves of QXN233. Bar = 2 cm.

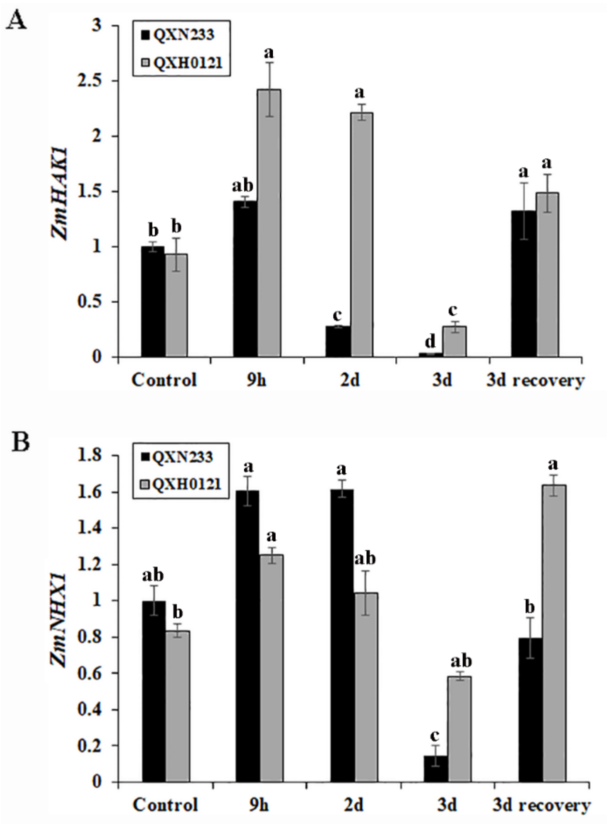

Figure S4. Expression analysis of *ZmHAK1* and *ZmNHX1* genes in QXN233 and QXH0121 roots under salt stress. The QXN233 and QXH0121 seedlings were exposed to 50 mM NaCl stress at 0 h, 9 h, 2 d, 3 d and recovery to normal condition for 3 d using a hydroponics assay, and the expression of two genes *ZmHAK1* (A) and *ZmNHX1* (B) in roots of them were examined by quantitative real-time PCR, respectively. Values represent means ± SE, and different letters indicate significant differences in QXH0121 vs QXN233 during the time of experiment (LSD test, $P < 0.05$).

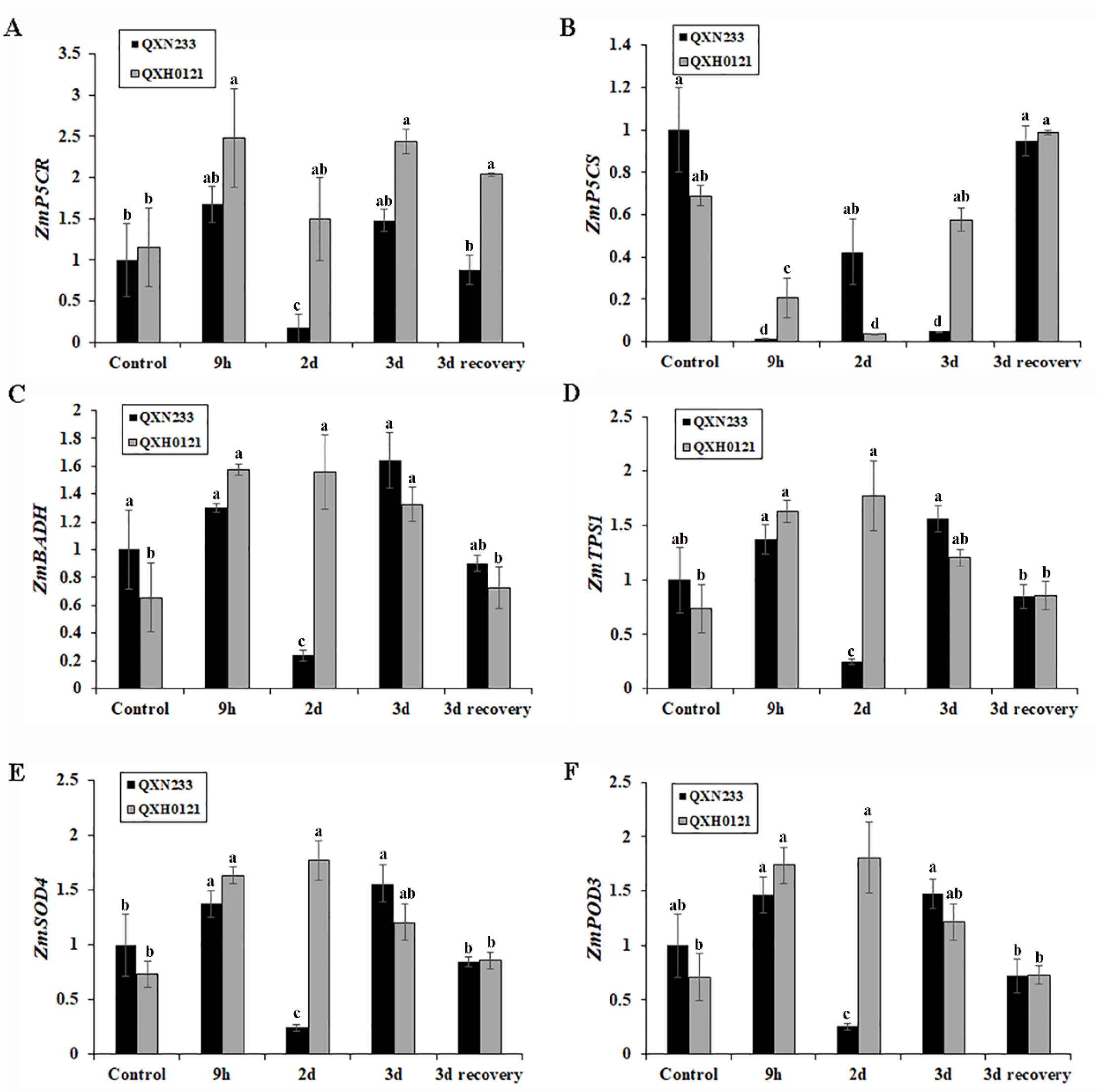

Figure S5. Expression analysis of *ZmP5CR*, *ZmP5CS*, *ZmBADH*, *ZmTPS1*, *ZmSOD4* and *ZmPOD3* genes in QXN233 and QXH0121 roots under salt stress. The QXN233 and QXH0121 seedlings were exposed to 50 mM NaCl stress at 0 h, 9 h, 2 d, 3 d and recovery to normal condition for 3 d using a hydroponics assay. *ZmP5CR* (A), *ZmP5CS* (B), *ZmBADH* (C), *ZmTPS1* (D), *ZmSOD4* (E) and *ZmPOD3* (F) genes in roots of them were examined by quantitative real-time PCR, respectively. Values represent means ± SE, and different letters indicate significant differences in QXH0121 vs QXN233 during the time of experiment (LSD test, $P < 0.05$).

Table S1. Primers used in quantitative real-time PCR.

Gene name	Genbank accession number	Forward/reverse primer	Sequence (5'-3')	Product (bp)
18S rRNA	AF168884	Forward	CCATCCCTCCGTAGTTAGCTTCT	151 bp
		Reverse	CCTGTCGGCCAAGGCTATATAC	
ZmNHX1	AY270036	Forward	ATGTGGCGTTACGGTGCGG	206 bp
		Reverse	GGTCATTTGGTGGGCGGG	
ZmHAK1	GRMZM2G093826	Forward	CGTGCCCACCAACCAGATGAC	123 bp
		Reverse	TTGATGAGTCGGGGGAACACG	
ZmP5CS	DQ864376	Forward	GCGAGGAAGTGGGCAAGTGGT	250 bp
		Reverse	TTGGGGAGGTGGGGTGGC	
ZmP5CR	DQ026301	Forward	CCAGCCTGTGCCAACCGC	147 bp
		Reverse	GTGCGGATGGCGGAGGC	
ZmBADH	EU019896	Forward	GGCTGTGCTGTTTTTTCTGGC	168 bp
		Reverse	GCCTTCTACGCCAGCAAATCTC	
ZmTPS1	AF529266	Forward	GGTTGCAGCGTTTCCTATTG	177 bp

		Reverse	AATCAAGAGATCGGTCCAGATG	
ZmSOD4	XM_008650839	Forward	TAAGCACCTGTGGCAACCGAT	115 bp
		Reverse	ACGAAACGGTCGGAATGCC	
ZmPOD3	GRMZM2G427815	Forward	TGACTGCTTTGTCCAGGGGTG	151 bp
		Reverse	GACTGCCTCCACCTGTGCCTT	
